# Inhibition of the Polyamine System Counteracts β-Amyloid Peptide-Induced Memory Impairment in Mice: Involvement of Extrasynaptic NMDA Receptors

**DOI:** 10.1371/journal.pone.0099184

**Published:** 2014-06-12

**Authors:** Guilherme Monteiro Gomes, Gerusa Duarte Dalmolin, Julia Bär, Anna Karpova, Carlos Fernando Mello, Michael R. Kreutz, Maribel Antonello Rubin

**Affiliations:** 1 Biochemistry and Molecular Biology Department, Natural and Exact Sciences Center, Federal University of Santa Maria, Santa Maria, RS, Brazil; 2 Research Group Neuroplasticity, Leibniz Institute for Neurobiology, Magdeburg, Germany; 3 Physiology and Pharmacology Department, Federal University of Santa Maria, Santa Maria, RS, Brazil; Nathan Kline Institute and New York University Langone Medical Center, United States of America

## Abstract

In Alzheimer's disease (AD), the β-amyloid peptide (Aβ) has been causally linked to synaptic dysfunction and cognitive impairment. Several studies have shown that N-Methyl-D-Aspartate receptors (NMDAR) activation is involved in the detrimental actions of Aβ. Polyamines, like spermidine and spermine, are positive modulators of NMDAR function and it has been shown that their levels are regulated by Aβ. In this study we show here that interruption of NMDAR modulation by polyamines through blockade of its binding site at NMDAR by arcaine (0.02 nmol/site), or inhibition of polyamine synthesis by DFMO (2.7 nmol/site), reverses Aβ_25–35_-induced memory impairment in mice in a novel object recognition task. Incubation of hippocampal cell cultures with Aβ_25–35_ (10 µM) significantly increased the nuclear accumulation of Jacob, which is a hallmark of NMDAR activation. The Aβ-induced nuclear translocation of Jacob was blocked upon application of traxoprodil (4 nM), arcaine (4 µM) or DFMO (5 µM), suggesting that activation of the polyamine binding site at NMDAR located probably at extrasynaptic sites might underlie the cognitive deficits of Aβ_25–35_-treated mice. Extrasynaptic NMDAR activation in primary neurons results in a stripping of synaptic contacts and simplification of neuronal cytoarchitecture. Aβ_25–35_ application in hippocampal primary cell cultures reduced dendritic spine density and induced alterations on spine morphology. Application of traxoprodil (4 nM), arcaine (4 µM) or DFMO (5 µM) reversed these effects of Aβ_25–35_. Taken together these data provide evidence that polyamine modulation of extrasynaptic NMDAR signaling might be involved in Aβ pathology.

## Introduction

Alzheimer's disease (AD) is the most frequent form of dementia in the elder population [1]. It is characterized by a progressive decline of cognitive function and accumulation of neurofibrillary tangles, formed by phosphorylated *tau* protein and senile plaques formed by amyloid-β-peptide (Aβ) accumulation [2]. Recent evidence suggests that the toxic effects of Aβ may be mediated, in part, by activation of extrasynaptic NMDARs [3], [4], although the mechanisms by which Aβ induces synaptic and memory impairment are not fully understood.

Polyamines, such as spermidine and spermine, are aliphatic amines that function as positive modulators of NMDAR. They bind at the lower lobe of the N-terminal domain of the GluN1 and GluN2B dimer interface, modulating agonist binding [5]. Upregulation of the polyamine system has been reported both in p*ost-mortem* analysis of AD's brain and *in vitro* studies. Polyamine levels were found increased in memory-related brain areas, like temporal cortex and frontal lobe [6], [7]. Also, addition of Aβ peptide to neuronal cell cultures increases polyamine levels, leading to NMDAR activation [8], [9]. In addition, increased ornithine decarboxylase (ODC) activity and immunostaining were reported in the brain of Alzheimer's disease patients AD and AD-like conditions [10], [11]. Although up-regulation of polyamine system in AD and AD-like conditions were reported, it is unclear whether these alterations are linked to Aβ-induced synaptic dysfunction and cognitive decline.

Here we investigated whether inhibition of polyamine system counteracts the cognitive impairment induced by Aβ_25–35_ in mice. Moreover, we tested whether inhibition of polyamine system could reverse the Aβ-induced alterations in extrasynaptic NMDAR activity and dendritic spine density and morphology. We show evidence that inhibition of polyamine system reverses the memory impairment induced by Aβ_25–35_, probably through relief of extrasynaptic NMDAR stimulation, which ultimately leads to spine pathology and cognitive impairment.

## Materials and Methods

### 3.1 Ethics Statement

All animal experimentation reported in this study was approved by the Local Ethics Committee – Comissão de Ética no Uso de Animais (process number 0206) and performed in accordance with the ARRIVE guidelines for animal experimentation [12], the Policies on the Use of Animals and Humans in Neuroscience Research, revised and approved by the Society for Neuroscience Research.

### 3.2. Behavioral experiments

#### 3.2.1. Subjects

Adult male Swiss mice (n = 163), approximately 12 weeks old (30–40 g), provided by the Animal Center of Universidade Federal de Santa Maria, were used for the behavioral experiments. They were housed 4 to 8 in plastic non-transparent cages, with free access to water and food (Guabi, Santa Maria, Rio Grande do Sul, Brazil), under controlled 12 h/12 h light-dark cycle (lights on at 07:00) conditions and temperature (24°C). Behavioral experiments were conducted in a sound-attenuated and air-regulated room, where the animals were habituated 1 hour prior to experiments. All possible means were applied to minimize animal suffering and to reduce the number of animals used.

#### 3.2.2. Drugs and treatments

N-[3-aminopropyl]-1,4-butanediamine trihydrochloride (spermidine), DL-α-difluoromethilornithine hydrochloride (DFMO), N-methyl D-aspartate (NMDA), Aβ_25–35_ and Aβ_35–25_ were purchased from Sigma (St. Louis, MO, USA); 1,4-diguanidinobutane sulfate (arcaine) was obtained from Pfaltz & Bauer (Waterbury, CT, USA); CP-101,606 (Traxoprodil) was kindly donated by Pfizer Inc. (New York, NY, USA). For behavioral experiments, traxoprodil, DFMO, arcaine and spermidine were dissolved in 50 mM phosphate buffer saline (PBS), pH 7.4.

Aβ_25–35_ and Aβ_35–25_ were dissolved in saline at a concentration of 3 mM and stored at −20°C. Aggregation of Aβ_25–35_ was performed following protocol described elsewhere [13]. Briefly, Aβ_25–35_ and Aβ_35–25_ peptide were dissolved in sterile bidistilled water at a concentration of 3 mM and stored at −20°C. Aβ_25–35_ and its inverted sequence were incubated at 37°C for 4 days in order to induce aggregation.

In all behavioral experiments, in order to deliver the treatments directly to the mice brain, Aβ_25–35_, Aβ_35–25_, spermidine, traxoprodil, arcaine and DFMO, were administered through intracerebroventricular (i.c.v.) route, according to Dalmolin and coworkers [14]. Briefly, mice were anesthetized with isofluorane (nasal route) until full anesthesia was achieved. The microinjections were performed using a Hamilton 10 µl syringe connected to a specially made 28-gauge stainless steel needle with 3 mm in length. The needle was inserted directly through the skin and skull into the lateral ventricle, targeted by visualizing an equilateral triangle between the eyes and center of skull to locate bregma, then inserting the needle 1 mm laterally to this point. This avoids the use of unnecessary force since the needle penetrates at the suture line of the skull plates. Compounds were injected in a volume of 3 µl over a 5 sec period, followed by a 10 sec delay to allow diffusion and prevent backflow. All injections were performed by an experimenter well trained in this technique. When co-administered (spermidine and arcaine), drugs were injected using a polyethylene tube attached to the Hamilton syringe. A bubble of 1 cm kept the drugs apart and an interval of approximately 30 seconds separated each drug injection.

#### 3.2.3. Novel Object Recognition Task

Novel object recognition task was performed in a 30×30×30 cm wooden chamber with walls painted black, the front wall made of Plexiglas and the floor covered with ethyl vinyl acetate sheet. A light bulb, hanging 60 cm above the behavioral apparatus, provided constant illumination of about 40 lux, and an air-conditioner provided constant background sound isolation. The objects used were plastic mounting bricks, each of them with different shapes and colors, but same size. Throughout the experiments objects were used in a counterbalanced manner and animals showed no preference for any of the objects. Chambers and objects were thoroughly cleaned with 30% ethanol before and after each animal was test.

**Figure 1:**
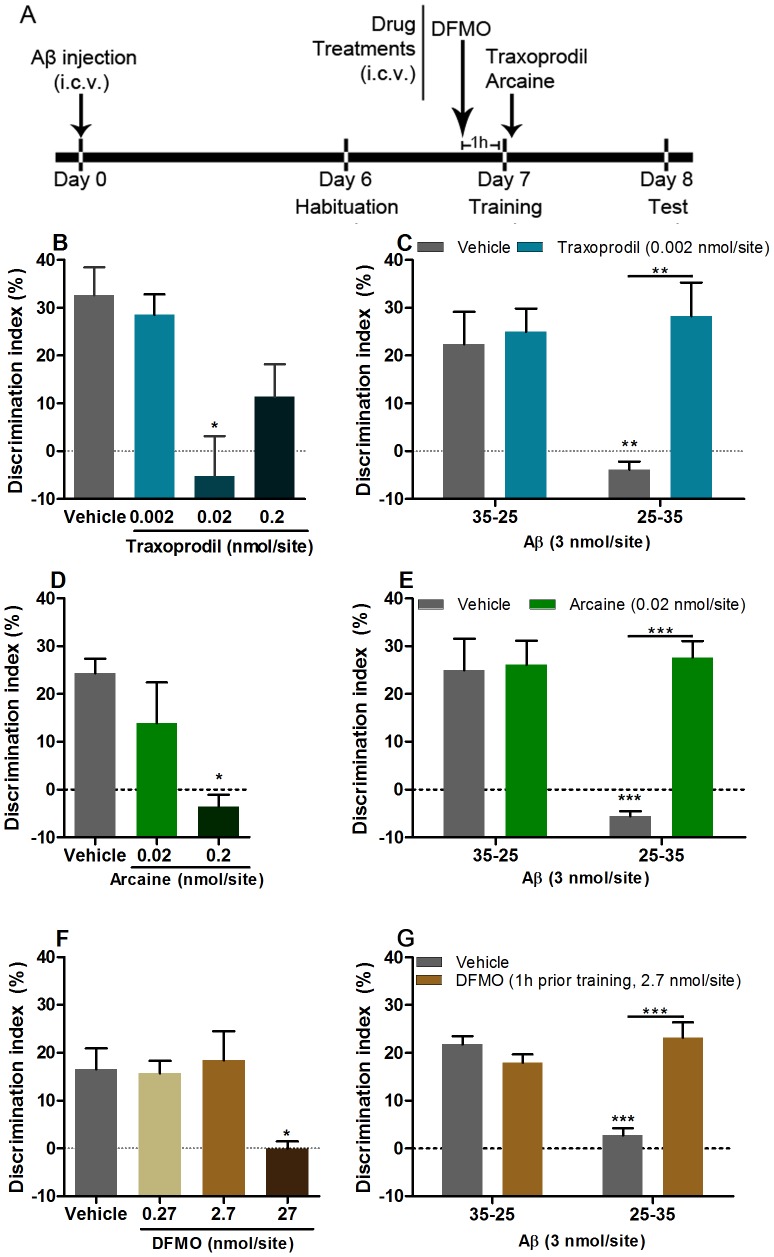
Traxoprodil (B, C), arcaine (D, E) and DFMO (F, G) restore memory of Aβ_25–35_-injected mice, in the novel object recognition task. (A) Experimental schedule, i.c.v., intracerebroventricular. Traxoprodil and arcaine were administered immediately after training. DFMO was administered 1 hour prior training. Data shown as mean +S.E.M. N = 3  = –5 animals per group for B, D, F. N = 5–9 animals per group for C, E, G. *P<0.05 when compared to control, **P<0.01 and *P*<0.001when compared to control or Aβ_25–35_ group.

**Figure 2:**
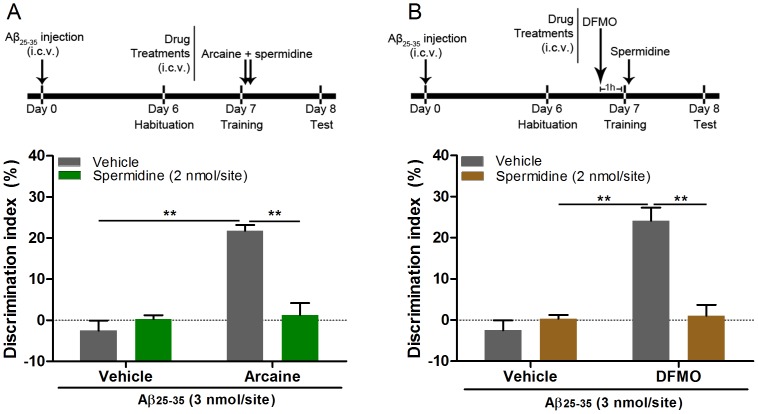
Spermidine administration reverses the ameliorative effect of arcaine and DFMO on memory of Aβ_25–35_-injected mice. (A) Spermidine was co-administered with arcaine (0.02 nmol/site) immediately after training. (B) (2.7 nmol/site) DFMO was administered 1 hour prior training and immediately after training were injected with spermidine. I.c.v.  =  intracerebroventricular. Data shown as mean + S.E.M. N = 6–7 per group. **p<0.001 when compared vehicle + arcaine or vehicle + DFMO control.

Six days after Aβ_25–35_ or Aβ_35–25_ injection the novel object recognition task was performed according to Wang and coworkers [15], with minor modifications. The task consisted of habituation, training and testing sessions, each of them lasting 8 minutes. In the first session, mice were habituated to the behavioral apparatus, with no objects, and then returned to their home cages. Twenty-four hours later, training session took place, where animals were exposed to two equal objects (object A), and the exploration time was recorded with two stopwatches. Exploration was recorded when the animal touched or reached the object with the nose at a distance of less than 2 cm. Climbing or sitting on the object was not considered exploration. Immediately after training the animals were randomly assigned to the following groups: vehicle (PBS 3 µl), traxoprodil (0.002–0.2 nmol/site), arcaine (0.02–0.2 nmol/site) or spermidine (2 nmol/site). DFMO (0.27–27 nmol/site) was given 1 hour prior training. Doses used were based on previous work [16], [17], [18] and dose-effect curves (Figure 1B, 1D, 1E). Figure 1A, 2A and 2B display a time-line with treatments and administration time. The test session was carried out 24 hours after training, when mice were placed back in the behavioral chamber and one of the familiar objects (i.e. object A) was replaced by a novel object (i.e. object B). The time spent exploring the familiar and the novel object was recorded. The discrimination index was then calculated, taking into account the difference of time spent exploring the new and familiar objects, ([(T_novel_ – T_familiar_)/(T_novel_ + T_familiar_)] ×100 (%)), and used as a memory parameter. Experiments and data analysis were conducted by an experimenter blind to treatment conditions.

### 3.3. *In vitro* experiments

#### 3.3.1 Primary hippocampal cell culture

Hippocampal primary cultures were prepared as described previously [19] using 19 days old Wistar rat embryos (Leibniz Institute for Neurobiology, breeding stock). Cells were plated in a density of 40.000 cells per 18 mm coverslips, grown in 1 ml of neurobasal medium (NB, Gibco) supplemented with B27 (Life Technologies) and 200 mM L-glutamine. Cells were kept in a humidified 95% air, 5% CO_2_ incubator at 37°C with no further change.

#### 3.3.2. Extrasynaptic NMDAR-induced Jacob nuclear accumulation

In order to assess extrasynaptic NMDAR activation and subsequent Jacob protein accumulation in the nucleus, assays were performed according to Behnisch and coworkers [20]. This protocol effectively induces non-phosphorylated Jacob accumulation in the nucleus, with concomitant reduction of Jacob levels at the dendritic shaft [21]. Hippocampal neurons (21 DIV) were successively incubated with 50 µM bicuculline (Tocris), 2.5 mM 4-AP (Tocris), in order to induce synaptic activity, and 10 µM MK-801 (Tocris) for irreversible blocking of activated synaptic NMDAR. After 30 minutes of incubation, cells were washed with conditioned NB medium and then 200 µM NMDA, (washed out after 3 minutes), or spermidine (400 µM), Aβ_25–35_ (10 µM), traxoprodil (4 nM), arcaine (4 µM), DFMO (5 µM) or the combination of Aβ_25–35_ + traxoprodil, Aβ_25–35_ + arcaine, Aβ_25–35_ + DFMO were applied for 30 minutes prior fixation. Spermidine, traxoprodil, arcaine and DFMO were dissolved in DMSO (final concentration of 0.001%). Anisomycin-containing medium (7.5 µM) was used throughout the experiments in order to block *de novo* protein synthesis.

Primary antibodies were diluted in blocking buffer (rabbit anti-pan-Jacob 1∶350) and incubated overnight at 4°C. After PBS washing, cells were incubated with fluorescent Alexa fluor 488 tagged secondary antibody (1∶1000; Molecular probes) for 60 min. Thereafter the cells were stained with DAPI (1∶1000, 10 min) in PBS and mounted on slides with Mowiol.

#### 3.3.3. Analysis of dendritic spine density and morphology

In order to perform analysis of dendritic spines morphology, hippocampal primary cultures were transfected with eGFP expressing plasmid at 7/9 DIV using Lipofectamine 2000 (Life Technologies). Briefly, 1.8 µg of eGFP-N1 plasmid (Clontech, Mountain View, CA) was mixed with 3 µl Lipofectamine 2000 in 200 µl of NB, incubated for 30 min, added to the neurons and incubated at 37°C in 5% CO_2_ for additional 60 min. Next, NB medium containing transfection mix was exchanged with conditioned NB and kept for additional 2 weeks at 37°C in 5% CO_2_.

At DIV 21, cells were incubated with Aβ_25–35_ (10 µM) for 24 hours. Two hours prior to 4% (w/v) paraformaldehyde fixation, traxoprodil (4 nM), arcaine (4 µM) or DFMO (5 µM) were mixed in the culture media. GFP expressing cultured neurons were washed, permeabilized with 0.2% Triton X-100 in PBS for 10 minutes washed with PBS and mounted on the slides with Mowiol. Drug concentration and application duration were chosen based on previous work [8], [9].

#### 3.3.4. Confocal laser scan microscopy

Images were acquired using a confocal microscope (Leica TCS-SP5-II. Leica-Microsystems, Mannheim, Germany) equipped with Plan Apo 63× oil NA 1.4 objective and a Diode (405 nm), Argon (458, 476, 488, 496, 514 nm laser lines), Diode Pumped Solid State (DPSS, 561 nm) and HeNe (633 nm) lasers. Laser intensity and signal detection settings were held constant to allow quantitative comparison between experimental groups. Cells were scanned with optical serial sections of 0.29 µm intervals and, depending on the experiment, maximum intensity or mean average projection of z-stack images from individual cell nuclei and dendritic branches were generated (ImageJ software, NIH, Bethesda, USA).

For dendritic spines density and morphology experiments, one to three dendritic segments (20 µm), distal and proximal from soma of each neuron were used, and analysis was performed using NeuronStudio software 0.9.92 [22]. Spines were defined as protrusions that could be differentiated from the dendritic shaft and restricted to those that were visible in the *x*- and *y*-axes.

Quantification of nuclear accumulation of Jacob was performed as previously described [19]. Briefly, images were open on Image J software and the nuclear region of interest (ROI) was defined using the threshold from the DAPI staining. Nuclear Jacob immunoreactivity was measured as mean grey values (arbitrary units in pixel intensity). Data plotted in the graphs were normalized relative to control group.

### 3.4. Statistical analysis

Statistical analysis was performed using GraphPad Prism Version 5.01. Values are given as mean ±S.E.M. One-way or two-way analysis of variance (ANOVA) was performed, followed by the Student-Newman-Keuls (SNK) test, depending on the experiment. Values of *P*<0.05 were considered significant.

## Results

### 4.1. Blockade of the polyamine binding site at NMDAR or inhibition of polyamine synthesis reverses Aβ_25–35_–induced memory impairment

In order to test whether inhibition of the polyamine system counteracts memory impairment induced by Aβ injection, mice injected with Aβ_25–35_, or its inverted sequence were treated with traxoprodil, a GluN2B antagonist of NMDAR, arcaine, an antagonist of the polyamine binding site at NMDAR, or DFMO, a polyamine synthesis inhibitor. We found no significant difference in the amount of time that animals of all groups spent exploring both objects in the training session, indicating no biased exploration of the objects (data not shown). However, during the test session, Aβ_25–35_-injected mice performed worse than controls in the novel object recognition task, as shown by a decrease in the discrimination index when compared to control (*P*<0.05, Fig. 1C, 1E, 1G). Of note, novel object recognition it is an interesting task in the study of Alzheimer's cognitive decline, since impairment of recognition memory is one of the first cognitive deficits present in AD patients [23].

Administration of traxoprodil (0.02 nmol/site) in naive mice significantly reduced the discrimination index for the novel object when compared to control (One-way ANOVA, F_(3,15)_ = 6.736, *P<*0.01 Fig. 1B). Administration of a dose of traxoprodil that had no effect in control mice (0.002 nmol/site) restored memory of Aβ_25–35_–injected mice, as indicated by a higher discrimination index when compared to the vehicle treated-Aβ_25–35_-injected group in the test session (Two-way ANOVA, F_(1,16)_ = 6.303, *P*<0.05, Fig. 1C).

Administration of arcaine (0.2 nmol/site) in naive mice significantly reduced the discrimination index for the novel object when compared to control (One-way ANOVA, F_(2,6)_ = 6.705, *P<*0.05 Fig. 1D). Administration of a dose that had no effect *per se* (0.02 nmol/site), restored memory in Aβ_25–35_ – injected mice, with a higher discrimination index when compared to the saline treated - Aβ_25–35_ -injected group in the test session (Two-way ANOVA, F_(1,16)_ = 18.91, *P*<0.001, Fig. 1E), suggesting that the polyamine binding site at NMDAR plays a role in the cognitive deficits induced by Aβ_25–35_.

We next administered the ODC enzyme inhibitor DFMO in order to verify whether blockade of enzymatic activity, and thereby blockade of polyamine synthesis, could rescue memory deficits of mice injected with Aβ_25–35_. DFMO (27 nmol/site) injected 1 hour prior training significantly reduced the discrimination index, compared to control mice treated with saline (One-way ANOVA, F_(3,8)_ = 4.44, *P<*0.05 Fig. 1F). The administration of DFMO, at a dose that had no effect in control mice (2.7 nmol/site), restored memory of animals injected with Aβ_25–35_ (Two-way ANOVA, F_(1,25)_ = 24.44, *P*<0.001, Fig 1G).

Since both arcaine and DFMO rescued memory deficits in mice injected with Aβ_25–35_, the next set of experiments was designed to assess whether the co-administration of spermidine could reestablish the memory impairment induced by Aβ_25–35_ injection, after it had been reversed by arcaine or DFMO. Spermidine (2 nmol/site), administrated immediately after training in arcaine-treated animals, significantly reduced the discrimination index (Two-way ANOVA, F_(1,22)_ = 72.09, *P*<0.0001, Fig. 2B). In a second experiment, mice injected with Aβ_25–35_ received DFMO 1 hour prior to training and then immediately after training spermidine was administered. This protocol reversed the ameliorative effect of DFMO on memory of mice injected with Aβ_25–35_ (Two-way ANOVA, F_(1,23)_ = 69.39, *P*<0.0001, Fig.2B). This data suggest that spermidine levels can influence whether learning is acquired or not by Aβ_25–35_-injected mice.

The *in vivo* protocol included treatment 7 days after Aβ injection, was designed in order to modulate memory consolidation in Aβ_25–35_-injected mice and to test therapeutic potential. The behavioral data suggest that Aβ_25–35_-injected animals, despite possible neurodegeneration, were capable to acquire new memories, as depicted in Figures 1C, 1E, 1G, 2A, 2B.

Dendritic spines are dynamic structures, and changes in their density and morphology, which can take place in minutes [24], [25], might underlie the memory improvement effect seen on the behavior experiments. Moreover, since long-term memory storage relies on structural plasticity [26], we designed an *in vitro* protocol that could mimic the *in vivo* approach used.

### 4.2. Blockade of the polyamine binding site at NMDAR and inhibition of polyamine synthesis reduce Aβ_25–35_–induced changes in spine number and morphology

**Figure 3:**
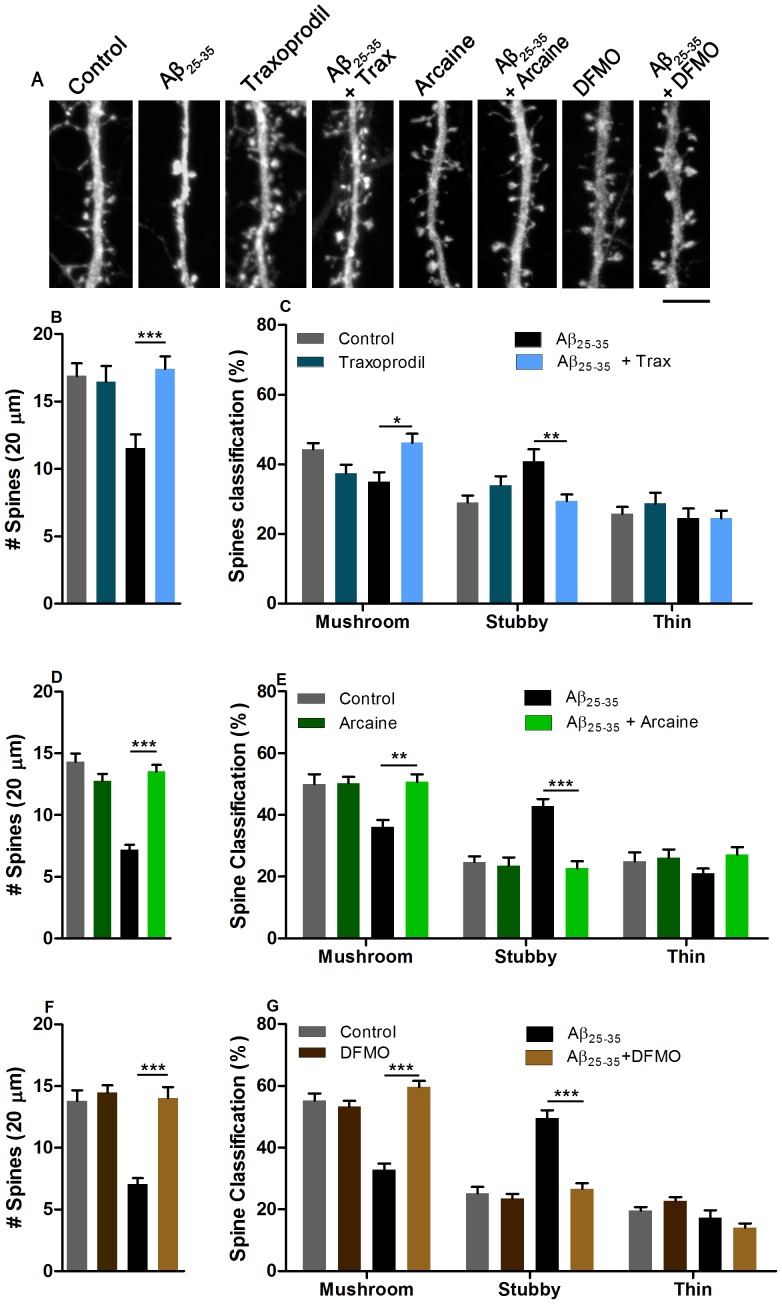
Traxoprodil (B, C), arcaine (D, E) or DFMO (F, G) rescues dendritic spine number and morphology changes induced by Aβ_25–35_ in hippocampal neuron cultures. (A) Representative micrographs depicting GFP-filled dendrites. Cells were incubated with Aβ_25–35_ for 24 hours and traxoprodil (4 nM), arcaine (4 µM) or DFMO (5 µM) were added 2 hours prior fixation. Spine density and morphology analysis was performed in 20 µm dendrite segments. Spines were classified according to their morphology. Scale bar represent 5 µm. In each experiment 30 to 48 dendritic segments were analyzed Data shown as mean + S.E.M. *P<0.05, **P<0.001, ***P<0.0001 compared to control group or as depicted in graphs.

Morphological consequences of Aβ pathology can be readily observed in neuronal primary cultures. We therefore next asked whether the effects of traxoprodil, arcaine or DFMO are correlated with changes in spine number and morphology. Incubation of hippocampal neurons for twenty-four hours with Aβ_25–35_ (10 µM) significantly decreased the number of dendritic spines (*P*<0.05, Fig. 3A, Fig. 3B, 3D, 3F) and markedly reduced the number of mushroom-like spines and induced a relative increase in stubby-like spines (*P*<0.05, Fig. 3C, 3E, 3G). Incubation of primary hippocampal neurons with traxoprodil (4 nM) for two-hours significantly rescued the decrease of spine number induced by Aβ_25–35_ (Two-way ANOVA, F_(1,143)_ = 9.220, *p* = 0.0028, Fig. 3B). Traxoprodil also rescued the Aβ_25–35_-induced changes in dendritic spine morphology with an increased number of mushroom-like spines and a reduction in stubby-like spines (Two-way ANOVA, F_(3,139)_ = 9.634, *p*<0.0001, Fig. 3C).

Blockade of the polyamine binding site at NMDAR with arcaine also attenuated the effect of Aβ_25–35_ application on dendritic spine morphology and number. Application of arcaine for two hours significantly blocked the Aβ-induced reduction spine number (Two-way ANOVA, F_(1,115)_ = 54.22, p<0.0001, Fig. 3D) and increased the number of mushroom like spines and reduced stubby like spines in cultures incubated with Aβ_25–35_, (Two-way ANOVA, F_(3,116)_ = 29.12, p<0.0001, Fig. 3E). Furthermore, inhibition of ODC by DFMO also rescued spine number (Two-way ANOVA, F_(1,123)_ = 18.15, p<0.0001, Fig. 3F) and morphology (Two-way ANOVA, F_(3,126)_ = 74.6, *p*<0.0001, Fig. 3G) of neurons incubated with Aβ_25–35_.

### 4.3. Blockade of the polyamine binding site at NMDARs or inhibition of polyamine synthesis abolish Aβ_25–35_-induced Jacob nuclear accumulation

Extrasynaptic NMDAR activation has been shown to mediate nuclear translocation of Jacob, which is followed by dephosphorylation of CREB, stripping of synaptic contacts, a simplification neuronal cytoarchitecture and eventually cell death [19]. Previous studies suggest that application of Aβ_1–42_ drives Jacob into the nucleus and that this depends upon activation of extrasynaptic GluN2B containing NMDARs [3]. To test whether inhibition of polyamine system also can block nuclear accumulation of Jacob induced by Aβ_25–35_, hippocampal neurons in culture were stimulated according to previously published protocols [20] and the nuclear accumulation of Jacob was determined by immunocytochemistry.

**Figure 4:**
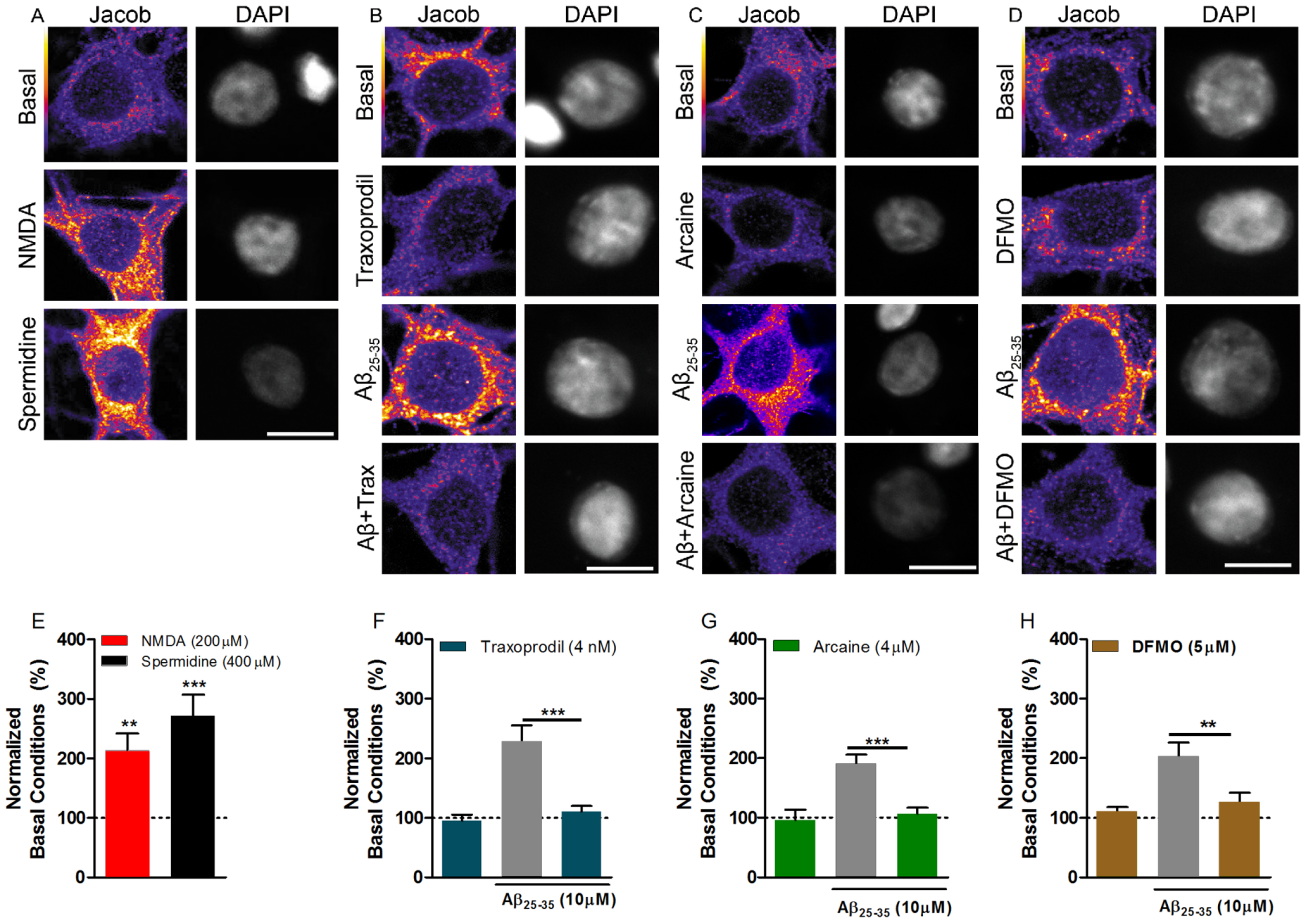
Spermidine and Aβ_-25–35_ induces nuclear Jacob accumulation of hippocampal neurons through stimulation of extrasynaptic NMDARs. NMDA (200 µM) and spermidine (400 µM) increases nuclear Jacob localization (A, E). Aβ_25–35_-induced Jacob translocation to the nucleus it is blocked by co-incubation with traxoprodil (4 nM) (B, F), or arcaine (C, G). Incubation of DFMO (5 µM) 10 min prior addition of Aβ_25–35_, also block Jacob translocation (D, H). Scale bars represents 10 µm. Data shown as mean + S.E.M., N: 17–24 cells per group. **p<0.001 and ***p<0.0001 compared to control group or as depicted in graphs.

Bath application of 100 µM NMDA for 3 min resulted in increased Jacob nuclear immunofluorescence levels (Fig. 4A, 3E). Incubation of cells with spermidine (400 µM) alone also induced nuclear translocation of Jacob (One-way ANOVA, F_(2,40)_ = 16.05; p<0.0001, Fig. 4A, 4E). Furthermore, incubation of primary hippocampal neurons with Aβ_25–35_ (10 µM) also led to increased nuclear Jacob immunofluorescence (Fig. 4B, 4C, 4D, 4F, 4G, 4H). The co-administration of traxoprodil (4 nM) (Two-way ANOVA, F_(1,83)_ = 13.52, *p* = 0.0004, Fig. 4B, 4F) or arcaine (4 µM) (Two-way ANOVA, F_(1,65)_ = 10.24, *p* = 0.002, Fig. 4C, 4G) significantly blocked the Aβ-induced increase of Jacob nuclear immunofluorescence. Moreover, incubation of cells with DFMO (5 µM) for 10 min prior to Aβ application, significantly reduced nuclear translocation of Jacob (Two-way ANOVA, F_(1,75)_ = 8.262, p = 0.0052), Fig. 4D, 4H). These results suggest that the polyamine binding site at extrasynaptic NMDAR is involved in Aβ_25–35_ induced nuclear import of Jacob.

## Discussion

Changes in polyamine system were reported both in AD and AD-like conditions [6], [7], [8], [9], [11], [27]. This is the first report showing that inhibition of this system can counteract the memory impairment induced by Aβ injection in mice. Using behavioral and cellular readouts we could show that inhibition of the polyamine system counteracts the deleterious effect of Aβ_25–35_ on memory formation of mice tested in the novel object recognition task. Inhibition of the polyamine system also reversed the reduction in dendritic spine density changes in dendritic spine morphology induced by Aβ. Moreover, blockade of the polyamine binding site at NMDARs reversed the Aβ-induced increase in nuclear translocation of Jacob, a marker of Aβ-induced extrasynaptic NMDAR activation [3]. We propose that Aβ_25–35_ injection increases ODC activity [9], which then results in increased spermidine and spermine levels, enhanced activation of extrasynaptic NMDAR by polyamines, spine pathology and memory impairment.

Polyamines, like spermidine and spermine, are modulators of NMDAR. The binding site resides in the dimer interface formed by the lower lobes of GluN1/GluN2B N-terminal domains. This GluN1/GluN2B dimer can be found in two different states, a desensitized and an active state. Binding of polyamines stabilizes the dimer in the active state, thus favoring agonist binding [5], [28]. In this study we could show that inhibition of the polyamine system reversed the memory impairment of Aβ_25–35_-injected mice, a model of AD-like cognitive deficit [29]. Both traxoprodil and arcaine, antagonists of the GluN2B NMDAR subunit and the polyamine binding site at NMDAR, rescued memory deficits of Aβ-injected mice in the novel object recognition task. Of note, polyamine levels were found increased in the temporal cortex of AD patients [6], [7], a brain area that is involved in processing of episodic memory, one of the first cognitive function impaired in AD [30]. We also found that spermidine administration, in arcaine- or DFMO-treated Aβ_25–35_-injected animals restored the Aβ-induced memory impairment. Taken together, these data suggest that a polyaminergic modulation impacts learning in Aβ_25–35_-injected animals and inhibition of ODC enzyme activity, through DFMO administration, indeed reversed the memory impairment of Aβ_25–35_-injected mice. ODC is the rate-limiting enzyme in the polyamine pathway, and its activity is tuned by both polyamine levels and antizyme activity. Antizyme is a regulatory protein that binds to ODC and forms an inactive complex [31]. Mäkitie and coworkers [27] reported increased immunoreactivity of antizyme inhibitor (AZIN) in the hippocampus of AD patients, which may explain the increased ODC activity found in the brain of AD patients [11]. Indeed, AZIN and NMDA receptors were shown to be co-localized in hippocampal neurons [27], suggesting that AZIN regulates glutamatergic signaling, possibly by controlling local production of polyamines.

While glutamatergic dysfunction has been implicated in the etiology of AD, memantine, the only FDA-approved NMDAR antagonist for the treatment of moderate-to-severe AD, has limited success [32]. Memantine is a low affinity NMDAR channel blocker with strong voltage dependency and rapid unblocking kinetics [33]. This profile would allow to prevent the tonic pathological extrasynaptic NMDAR activation, while sparing physiological transmission [34], [35]. However, memantine's lack of selectivity over NMDAR, like antagonism of α7 nAChR [36], [37], might hamper its effectiveness in the treatment of AD. Thus, modulation of allosteric GluN2B NMDAR sites, e.g. polyamine binding site, could be a preferred strategy on the quest for AD's disease-modifying therapies. Allosteric modulation would confer several advantages: specific modulation of receptor subunits (like the GluN2B antagonist traxoprodil); allosteric modulators do not compete with physiological agonists and allosteric antagonism spare biological patterns of receptor activity [38], [39].

Several studies have shown that impairment of synaptic plasticity induced by Aβ peptide relies on NMDAR activation [40], [41], [42]. The location of this receptor might account for the toxic effects of its activation, once different compartmentalized signaling cascades are activated after synaptic or extrasynaptic NMDARs stimulation [43]. Activation of extrasynaptic NMDARs leads to translocation of Jacob to the nucleus, which is followed by reduction in dendritic spine density and simplification of dendritic tree [19], [21]. Aβ_1–42_ oligomers induce nuclear accumulation of Jacob and this translocation was blocked by the GluN2B antagonist ifenprodil [3]. Likewise, we found increased nuclear Jacob immunoreactivity upon spermidine or Aβ_25–35_ application in hippocampal cell cultures. The effect was abolished upon inhibition of the polyamine system and application of the GluN2B- antagonist traxoprodil, suggesting a similar mechanism like after application of Aβ_1–42_.

In AD, the reduction in synapses and dendritic spine number is strongly correlated with cognitive decline [44], [45] and changes in dendritic spine morphology are correlated with cognitive function. Thus, both spine loss and spine morphology changes may affect memory of Aβ_25–35_-injected mice. The incubation of hippocampal cell cultures with Aβ_25–35_ significantly reduced dendritic spine density and induced a reduction in mature spines (mushroom spines) and an increase in non-functional spines (stubby spines). Inhibition of the polyamine system, either by blockade of the polyamine binding site of NMDARs or inhibition of polyamine synthesis counteracted the deleterious effects of Aβ_25–35_ on dendritic spine number and morphology. Therefore, polyamines, through its action on NMDARs located at extrasynaptic sites could be involved in the loss of synaptic contacts and simplification of neuronal morphology that ultimately would lead to memory impairment.

We have shown that Aβ_25–35_ induced stripping of synaptic contacts, probably via stimulation of extrasynaptic NMDAR, a mechanism known to induce cell death [19], [46]. While we have no evidence to support the induction of neuronal cell death in the protocols used in this study, it has been suggested that early AD's cognitive impairment occurs in the absence of cell death [3], [47], mainly through disruption of synaptic transmission. Rönicke and coworkers [3] have shown that Aβ oligomers can induce synaptic contacts retraction and reduction of spontaneous network activity in cultured neurons without inducing cell death. Furthermore, it has been shown that sublethal concentrations of Aβ oligomers can impair long-term potentiation (LTP) induction [3], [42] enhance long-term depression (LTD) [48]. Noteworthy, Aβ-induced synaptic alterations results in disruption of neuronal network function [47], [49]. Disruption of normal cross-laminar cortical processing coincides with a decline of contextual fear learning [50], indicating that weakening of synaptic contacts might result in immediate disturbance of cognitive function. Together, these data suggests that, rather than cell death, synaptic transmission and structural plasticity disturbance might be pivotal to acute Aβ_25–35_ memory impairment.

Here, we reported that inhibition of polyamine system counteracted the Aβ_25–35_ – induced cognitive deficit in mice. Noteworthy, once established, both the memory impairment and the alterations in the neuronal morphology induced by Aβ_25–35_ were properly reversed by blockade of polyamine binding site at the NMDAR, possibly located at extrasynaptic sites, suggesting that modulation of this system might represent an attractive target for pharmacological interventions.

## References

[1] Price M, Jackson J (2009) World Alzheimer's Report. London. Alzheimer's Disease International.

[2] Roberson ED, Mucke L (2006) 100 years and counting: Prospects for defeating Alzheimer's disease. Science 3 314(5800): 781–784. 10.1126/science.1132813 PMC3544944 17082448

[3] Rönicke R, Mikhaylova M, Rönicke S, Meinhardt J, Schröder UH, et al (2011) Early neuronal dysfunction by amyloid β oligomers depends on activation of NR2B-containing NMDA receptors. Neurobiol Aging 32(12): 2219–2228. 20133015 10.1016/j.neurobiolaging.2010.01.011

[4] Talantova M, Sanz-Blasco S, Zhang X, Xia P, Akhtar MW, et al (2013) Aβ induces astrocytic glutamate release, extrasynaptic NMDA receptor activation, and synaptic loss. Proc Natl Acad Sci 2 110(27): E2518–27. 10.1073/pnas.1306832110 PMC3704025 23776240

[5] Mony L, Zhu S, Carvalho S, Paoletti P (2011) Molecular basis of positive allosteric modulation of GluN2B NMDA receptors by polyamines. EMBO Journal 30: 3134–3136. 21685875 10.1038/emboj.2011.203 PMC3160180

[6] Inoue k, Tsutsui H, Akatsu H, Hashizume Y, Matsukawa N, et al (2013) Metabolic profiling of Alzheimer's disease brains. Sci Rep 6(3)): 2364. 10.1038/srep02364 PMC3734482 23917584

[7] Morrison LD, Kish SJ (1995) Brain polyamine levels are altered in Alzheimer's disease. Neurosci. Lett 197: 5–8. 10.1016/0304-3940(95)11881-v 8545054

[8] Yatin SM, Yatin M, Aulick T, Ain KB, Butterfield DA (1997) Alzheimer's amyloid beta-peptide associated free radicals increase rat embryonic neuronal polyamine uptake and ornithine decarboxylase activity: protective effect of vitamin. E Neurosci Lett 263: 17–20. 10.1016/s0304-3940(99)00101-9 10218900

[9] Yatin SM, Yatin M, Varadarajan S, Ain KB, Butterfield DA (2001) Role of spermine in amyloid beta-peptide-associated free radical-induced neurotoxicity. J Neurosci Res 63: 395–401. 11223914 10.1002/1097-4547(20010301)63:5<395::AID-JNR1034>3.0.CO;2-Q

[10] Bernstein HG, Müller M (1995) Increased immunostaining for L-ornithine decarboxylase occurs in neocortical neurons of Alzheimer's disease patients. Neurosci Lett 17182(2–3): 123–126. 10.1016/0304-3940(95)11301-c 7777179

[11] Morrison LD, Cao XC, Kish SJ (1998) Ornithine decarboxylase in human brain: influence of aging, regional distribution, and Alzheimer's disease. J Neurochem 71: 288–294. 9648877 10.1046/j.1471-4159.1998.71010288.x

[12] Kilkenny C, Browne W, Cuthill IC, Emerson M, Altman DG, NC3Rs Reporting guidelines working group (2010) Animal research: reporting in vivo experiments: the ARRIVE guidelines. Br J Pharmacol 160(7): 1577–1579. 20649561 10.1111/j.1476-5381.2010.00872.x PMC2936830

[13] Maurice T, Lockhart BP, Privat A (1996) Amnesia induced in mice by centrally administered beta-amyloid peptides involves cholinergic dysfunction. Brain Res 706: 181–193. 8822355 10.1016/0006-8993(95)01032-7

[14] Dalmolin GD, Silva CR, Rigo FK, Gomes GM, Cordeiro Mdo N, et al (2011) Antinociceptive effect of Brazilian armed spider venom toxin Tx3-3 in animal models of neuropathic pain. Pain 152(10): 2224–2232. 21570770 10.1016/j.pain.2011.04.015

[15] Wang D, Noda Y, Zhou Y, Mouri A, Mizoguchi H, et al (2007) The allosteric potentiation of nicotinic acetylcholine receptors by galantamine ameliorates the cognitive dysfunction in beta amyloid_25–35_ i.c.v.-injected mice: Involvement of dopaminergic systems. Neuropsychopharmacology 32: 1261–1271. 17133263 10.1038/sj.npp.1301256

[16] Rubin MA, Berlese DB, Stiegemeier JA, Volkweis MA, Oliveira DM, et al (2004) Intra-amygdala administration of polyamines modulates fear conditioning in rats. J Neurosci 24(9): 2328–2334. 14999084 10.1523/JNEUROSCI.1622-03.2004 PMC6730445

[17] Gomes GM, Mello CF, da Rosa MM, Bochi GV, Ferreira J, et al (2010) Polyaminergic agents modulate contextual fear extinction in rats. Neurobiol Learn Mem 93(4): 589–595. 20206278 10.1016/j.nlm.2010.02.007

[18] Silva MA, Klafke JZ, Rossato MF, Gewehr C, Guerra GP, et al (2011) Role of peripheral polyamines in the development of inflammatory pain. Biochem Pharmacol 82(3): 269–277. 21570380 10.1016/j.bcp.2011.04.015

[19] Dieterich DC, Karpova A, Mikhaylova M, Zdobnova I, König I, et al (2008) Caldendrin-Jacob: A protein liaison that couples NMDA receptor signaling to the nucleus. PLOS Biol 6(2): 286–306. 10.1371/journal.pbio.0060034 PMC2253627 18303947

[20] Behnisch T, YuanXiang P, Bethge P, Parvez S, Chen Y, et al (2011) Nuclear translocation of Jacob in hippocampal neurons after stimuli inducing long-term potentiation but not long-term depression. PLoS ONE 6(2): e17276. 21364755 10.1371/journal.pone.0017276 PMC3041791

[21] Karpova A, Mikhaylova M, Bera S, Bär J, Reddy PP, et al (2013) Encoding and transducing the synaptic or extrasynaptic origin of NMDA receptor signals to the nucleus. Cell 28 152(5): 1119–1133. 10.1016/j.cell.2013.02.002 23452857

[22] Rodriguez A, Ehlenberger DB, Dickstein DL, Hof PR, Wearne SL (2008) Automated three-dimensional detection and shape classification of dendritic spines from fluorescence microscopy images. PLoS ONE 3(4): e1997. 18431482 10.1371/journal.pone.0001997 PMC2292261

[23] Perfecto K, Ahern NR (2013) Early assessment for Alzheimer's disease dementia: comparison of two metamemory diagnostic tests. J Psychosoc Nurs Ment Health Serv 51(9): 17–21. 10.3928/02793695-20130731-01 23938066

[24] Fischer M, Kaech S, Knutti D, Matus A (1998) Rapid actin-based plasticity in dendritic spines. Neuron 20: 847–854. 9620690 10.1016/s0896-6273(00)80467-5

[25] Dunaevsky A, Tashiro A, Majewska A, Mason C, Yuste R (1999) Developmental regulation of spine motility in the mammalian central nervous system. Proc Natl Acad Sci USA 96: 13438–13443. 10557339 10.1073/pnas.96.23.13438 PMC23966

[26] Kasai H, Fukuda M, Watanabe S, Hayashi-Takagi A, Noguchi J (2010) Structural dynamics of dendritic spines in memory and cognition. Trends Neurosci 33(3): 121–129. 20138375 10.1016/j.tins.2010.01.001

[27] Mäkitie LT, Kanerva K, Polvikoski T, Paetau A, Andersson LC (2010) Brain neurons express ornithine decarboxylase-activating antizyme inhibitor 2 with accumulation in Alzheimer's Disease. Brain Pathol 20(3): 571–580. 19832840 10.1111/j.1750-3639.2009.00334.x PMC8094758

[28] Kew JN, Kemp JA (1998) An allosteric interaction between the NMDA receptor polyamine and ifenprodil sites in rat cultured cortical neurons. J Physiol 412(1): 17–28. 10.1111/j.1469-7793.1998.017bf.x PMC2231188 9729614

[29] Kaminsky YG, Marlatt MW, Smith MA, Kosenko EA (2010) Subcellular and metabolism examination of amyloid-β peptides in Alzheimer disease pathogenesis: Evidence for Aβ_25–35_ . Exp Neurol 221: 26–37. 19751725 10.1016/j.expneurol.2009.09.005

[30] Dickerson BC, Eichenbaum H (2010) The episodic memory system: Neurocircuitry and disorders. Neuropsychopharmacology 35: 86–104. 19776728 10.1038/npp.2009.126 PMC2882963

[31] Coffino P (2001) Regulation of cellular polyamines by antizyme. Mol Cell Biol 2: 188–194. 10.1038/35056508 11265248

[32] Schneider LS, Dagerman KS, Higgins JP, McShane R (2011) Lack of evidence for the efficacy of memantine in mild Alzheimer's Disease. Arch Neurol 68(8): 991–998. 21482915 10.1001/archneurol.2011.69

[33] Danysz W, Parsons CG (2012) Alzheimer's disease, β-amyloid, glutamate, NMDA receptors and memantine – searching for the connections. Br J Pharmacol 167(2): 324–352. 22646481 10.1111/j.1476-5381.2012.02057.x PMC3481041

[34] Xia P, Chen HV, Zhang D, Lipton SA (2010) Memantine preferentially blocks extrasynaptic over synaptic NMDA receptor currents in hippocampal autapses. J Neurosci 30(33): 11246–11250. 20720132 10.1523/JNEUROSCI.2488-10.2010 PMC2932667

[35] Mota SI, Ferreira IL, Rego AC (2014) Dysfunctional synapse in Alzheimer's disease – A focus on NMDA receptors. Neuropharmacology 76(Pt A): 16–26. 10.1016/j.neuropharm.2013.08.013 23973316

[36] Aracava Y, Pereira EF, Maelicke A, Albuquerque EX (2005) Memantine blocks alpha7* nicotinic acetylcholine receptors more potently than n-methyl-D-aspartate receptors in rat hippocampal neurons. J Pharmacol Exp Ther 312(3): 1195–1205. 15522999 10.1124/jpet.104.077172

[37] Buisson B, Bertrand D (1998) Open-channel blockers at the human α4β2 neuronal nicotinic acetylcholine receptor. Mol Pharmacol 53: 555–563. 9495824 10.1124/mol.53.3.555

[38] Mony L, Kew JN, Gunthorpe MJ, Paoletti P (2009) Allosteric modulators of NR2B-containing NMDA receptors: molecular mechanisms and therapeutic potential. Br J Pharmacol 157(8): 1301–1317. 19594762 10.1111/j.1476-5381.2009.00304.x PMC2765303

[39] Paoletti P, Bellonec C, Zhou Q (2013) NMDA receptor subunit diversity: impact on receptor properties, synaptic plasticity and disease. Nat Rev Neurosci 14(6): 383–400. 23686171 10.1038/nrn3504

[40] Kamenetz F, Tomita T, Hsieh H, Seabrook G, Borchelt D, et al (2003) APP processing and synaptic function. Neuron 37: 925–937. 12670422 10.1016/s0896-6273(03)00124-7

[41] Lo D, Grossberg GT (2011) Use of memantine for the treatment of dementia. Expert Rev Neurother 11(10): 1359–1370. 21955192 10.1586/ern.11.132

[42] Shankar GM, Bloodgood BL, Townsend M, Walsh DM, Selkoe DJ, et al (2007) Natural oligomers of the Alzheimer amyloid-beta protein induce reversible synapse loss by modulating an NMDA-type glutamate receptor-dependent signaling pathway. J Neurosci 27: 2866–2875. 17360908 10.1523/JNEUROSCI.4970-06.2007 PMC6672572

[43] Hardingham GE. Bading H (2010) Synaptic versus extrasynaptic NMDA receptor signaling: implications for neurodegenerative disorders. Nat Rev Neurosci 11: 682–696. 20842175 10.1038/nrn2911 PMC2948541

[44] DeKosky ST. Scheff SW (1990) Synapse loss in frontal cortex biopsies in Alzheimer's disease: correlation with cognitive severity. Ann Neurol 27: 457–464. 2360787 10.1002/ana.410270502

[45] Walsh DM, Selkoe DJ (2004.) Deciphering the molecular basis of memory failure in Alzheimer's disease. Neuron 44: 181–193. 15450169 10.1016/j.neuron.2004.09.010

[46] Hardingham GE, Fukunaga Y, Bading H (2002) Extrasynaptic NMDARs oppose synaptic NMDARs by triggering CREB shut-off and cell death pathways. Nat Neurosci 5(5): 405–414. 11953750 10.1038/nn835

[47] Palop JJ, Mucke L (2010) Amyloid-beta-induced neuronal dysfunction in Alzheimer's disease: from synapses toward neural networks. Nat Neurosci 13(7): 812–818. 20581818 10.1038/nn.2583 PMC3072750

[48] Li S, Hong S, Shepardson NE, Walsh DM, Shankar GM, et al (2009) Soluble oligomers of amyloid beta protein facilitate hippocampal long-term depression by disrupting neuronal glutamate uptake. Neuron 62(6): 788–801. 19555648 10.1016/j.neuron.2009.05.012 PMC2702854

[49] Small DH (2008) Network dysfunction in Alzheimer's disease: does synaptic scaling drive disease progression? Trends Mol Med 3: 103–108. 10.1016/j.molmed.2007.12.006 18262842

[50] Lison H, Happel MF, Schneider F, Baldauf K, Kerbstat S, et al (2014) Disrupted cross-laminar cortical processing in β amyloid pathology precedes cell death. Neurobiol Dis 63: 62–73. 24291517 10.1016/j.nbd.2013.11.014

